# Characterization of the complete mitochondrial genome of *Odontobutis platycephala* collected from Nakdong River, South Korea

**DOI:** 10.1080/23802359.2019.1687362

**Published:** 2019-11-08

**Authors:** Md. Jobaidul Alam, Kyung Su Kim, Sapto Andriyono, Wongyu Park, Hae-Ja Baek, Jae-Young Je, Hyun-Woo Kim

**Affiliations:** a Interdisciplinary Program of Biomedical, Mechanical and Electrical Engineering, Pukyong National University, Busan, Republic of Korea;; b Gyeongsangnam-do Freshwater Fish Research Center, Miryang, Republic of Korea;; c Fisheries and Marine Faculty, C Campus Jl, Universitas Airlangga, Surabaya, Indonesia;; d Department of Marine Biology, Pukyong National University, Busan, Republic of Korea;; e Department of Marine-Bio Convergence Science, Pukyong National University, Busan, Republic of Korea

**Keywords:** Next-generation sequencing, *Odontobutis platycephala*, mitochondrial genome

## Abstract

The complete mitochondrial genome of *Odontobutis platycephala* collected from a native Korean river was determined by the bioinformatics assembly of the next-generation sequencing (NGS) reads. The circular mitogenome was 17,590 bp length which harbored canonical 13 protein-coding genes, 22 tRNAs, and 2 rRNAs, which was identical to those of family Odontobutidae. Twenty-eight genes were located on H strand, whereas remaining nine genes were on L strand. Except for COX1 gene (GTG), other 12 protein-coding genes were predicted typical start codons (ATG). Among the currently known mitogenome sequences, *O. platycephala* showed highest identity (96.98%) to Korean haplotype of *O. platycephala* (NC010199).

Fish in the genus *Odontobutis* are the freshwater sleepers native to East Asia and three species in the genus are currently reported in Korea; *Odontobutis platycephala* and *Odontobutis interrupta, and Odontobutis obscura* (Chae 1999; Iwata and Sakai 2002; Jun et al. 2016). We here report the complete mitochondrial genome of *O. platycephala,* which has been collected from a tributary of Nakdong River, South Korea (E128°06′27.73″, N35°32′20.96″) in 2018. The collected specimen and its DNA were stored at the Marine Biodiversity Institute of Korea (MABIK GR00002616). Its COI region showed 99.82% nucleotide sequence identity to the Korean haplotype of *O. platycephala* (JX679047). The complete mitochondrial DNA from the specimen was isolated by a commercially available kit (Abcam, Cambridge, MA, USA), then the complete mitochondrial genome was determined by the Illumina MiSeq sequencing method. The TruSeq^®^ RNA library preparation kit V2 (Illumina, San Diego, CA, USA) was used with the fragmented mitochondrial DNA by Covaris M220 Focused-Ultrasonicator (Covaris Inc., Woburn, MA, USA). The complete circular mitochondrial DNA was constructed by the bioinformatics assembly of the raw reads using Geneious software version 11.0.2 (Biomatters Ltd., Auckland, New Zealand)(Kearse et al. 2012). The secondary structures of 22 tRNAs were predicted by ARWEN program (Laslett and Canbäck 2008).

The complete circular mitogenome of *O. platycephala* (MN416814) was 17,590 bp in length, which consisted of 13 protein-coding genes, 22 tRNAs, and 2 ribosomal RNAs (12S and 16S). As shown in the other fish in the genus *Odontobutis*, the unusual additional non-coding region was identified between ND4 and ND5 as a result of a rearrangement of tRNAs between them (Ki et al. 2008). Total of 12 protein-coding genes was encoded on heavy strand (H), whereas ND6 was encoded on light strand (L). The typical control region (1287 bp) was identified between tRNA-*Pro* and tRNA-*Phe*, while the origin of light strand (O_L,_ 32 bp) was located between tRNA-*Asn* and tRNA-*Cys* at the WANCY tRNA cluster. Twelve protein-coding genes were started with typical ATG, whereas COX1 started with GTG. The incomplete stop codons (TA–/T––) were predicted in eight genes including *ND1*, *ND2*, *ATP6*, *COX2*, *COX3*, *ND3*, *ND4*, and *Cytb* genes.

A phylogenetic tree for the currently reported mitogenomes of Gobiiformes was constructed using the MEGA version 7 program with minimum evolution algorithm (Kumar et al. 2016). Among six mitogenomes in the same genus, *O. platycephala* collected from the Nakdong River showed the highest nucleotide sequence identity (96.98%) to the Korean haplotype of *O. platycephala* (NC010199), followed by the Chinese *Odontobutis yaluensis* (85.83%, NC022818) (Figure 1). It is noteworthy that a high degree of genetic distance between the same species in Korea and further biogeographical study should be made to have a better understanding of the evolutional relationship within the genus *Odontobutis*.

**Figure 1.**
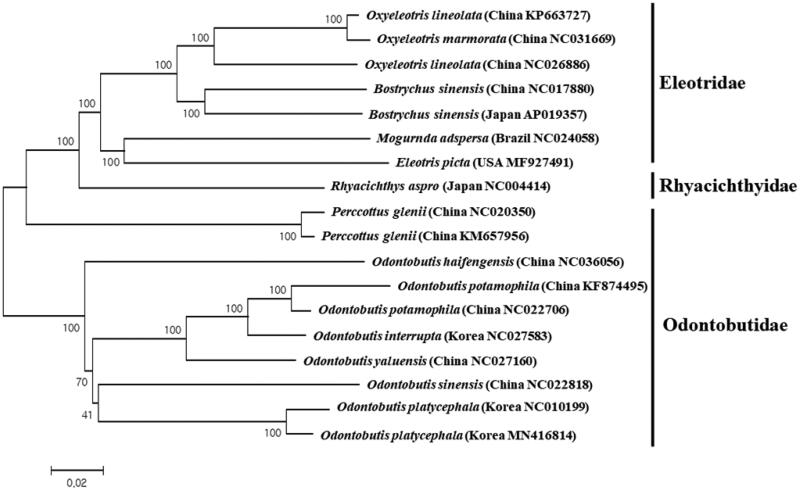
Phylogenetic relationship of *Odontobutis platycephala* in the order Perciformes. A phylogenetic tree was constructed with the currently reported complete mitogenomes in the order Perciformes by using the MEGA 7.0 software by Minimum Evolution (ME) algorithm with 1000 bootstrap replications. GenBank accession numbers and origin of the haplotypes were shown followed by each species scientific name.
